# In with the Old, in with the New: The Promiscuity of the Duplication Process Engenders Diverse Pathways for Novel Gene Creation

**DOI:** 10.1155/2012/341932

**Published:** 2012-09-13

**Authors:** Vaishali Katju

**Affiliations:** Department of Biology, University of New Mexico, Albuquerque, NM 87131, USA

## Abstract

The gene duplication process has exhibited far greater promiscuity in the creation of paralogs with novel exon-intron structures than anticipated even by Ohno. In this paper I explore the history of the field, from the neo-Darwinian synthesis through Ohno's formulation of the canonical model for the evolution of gene duplicates and culminating in the present genomic era. I delineate the major tenets of Ohno's model and discuss its failure to encapsulate the full complexity of the duplication process as revealed in the era of genomics. I discuss the diverse classes of paralogs originating from both DNA- and RNA-mediated duplication events and their evolutionary potential for assuming radically altered functions, as well as the degree to which they can function unconstrained from the pressure of gene conversion. Lastly, I explore theoretical population-genetic considerations of how the effective population size (*N*_*e*_) of a species may influence the probability of emergence of genes with radically altered functions.

## 1. Introduction

A recognition of the significance of novel traits for the origin of biological complexity and diversity is not new. Darwin himself spelled out the link between the evolution of novel traits and origin of new life forms, despite an agonizing lack of awareness of the genetic nature of variation and heredity. Armed with knowledge of the molecular basis of biological inheritance stemming from the rediscovery of Mendelian genetics and the first cytological glimpses of duplication events [1, 2], neo-Darwinists and early geneticists were swift to recognize the evolutionary potential of gene duplication as a means of exapting ancestral genes for novel functions [3–5]. The evolutionary advantage of gene duplication was appreciated well in advance of the discovery of DNA and was well surmised by Huxley in 1942—“… small repeats of this type … constituted the chief method by which the number of genes is increased, thus providing duplicate factors and the opportunity for slight divergent specialization of homologous genes, giving great delicacy of adjustment” [6]. Gene duplication research in the 1940s through 1950s was decidedly cytological in flavour, often employing mutagenic treatments to accelerate mutation rates for the purpose of identifying the frequency, chromosomal location, and breakpoints of duplications and other structural variants. ([7–12], among others). Commencing in the early 1960s, experimental studies of gene duplication took on more of an evolutionary perspective, with greater efforts being directed at the nature of molecular evolutionary change due to alterations of the base composition, and the identification of different types of duplications leading to novel genes with radically altered reading frames [13–15]. Notably, a few studies specifically identified the creation of *partial* gene duplicates by incomplete duplication of the progenitor copy's open reading frame as in the case of human haptoglobins [13], human hemoglobins [14], and protamines in the Pacific herring *Clupea pallasi* [15], among others. Indeed, in their article, Smithies et al. [13] succinctly detailed the evolutionary potential of such radically altered gene duplicates—“We suggest that proteins with radically changed properties can be formed as a consequence of the single genetic event of a chromosomal rearrangement involving non-integral numbers of genes. Chromosomal rearrangements of this type appear to provide a mechanism for achieving more rapid and extensive changes in protein structure in evolution than are possible by point mutations even when preceded by gene duplication.” However, a true recognition of the role of gene duplication in the creation of radically altered structures would not be forthcoming until the advent of the genomic revolution.

Susumu Ohno is largely credited with formalizing and instigating the study of gene duplication into the burgeoning field it is today with the publication of his treatise titled *Evolution by Gene Duplication* [16]. In his book, Ohno hypothesized that the vertebrate lineage had undergone two rounds of whole-genome duplication; variations of his idea are now collectively referred to as the “two rounds” (2R) hypothesis (e.g., [17–19]). Although modest in size and somewhat simplistic and narrow in its depiction of the plausible pathways of gene duplication, *Evolution by Gene Duplication* has certainly earned its keep as the first book entirely devoted to the subject of gene and genome duplication. It also provided the first theoretical framework for the evolution of novel gene function by one copy following gene duplication. Ohno postulated that single-copy genes with essential functions are actively policed by purifying natural selection that serves to eliminate newly-acquired “forbidden” mutations that may compromise the ancestral gene function. This active removal of new mutations by single-copy genes in turn precludes them from exploring new evolutionary space (and gain of novel functions). The gene duplication process, by creating a redundant locus, simultaneously (i) permits the uninterrupted maintenance of the ancestral function by one copy and (ii) enables the extra, initially redundant copy to accumulate mutations that facilitate its rebirth as a new gene with a “hitherto non-existent function” (neofunctionalization) or hasten its degeneration into a “nonsense, DNA base sequence” [16, 20] or pseudogene (nonfunctionalization).

Analyses of entire populations of young gene duplicates identified from whole-genome sequence data have established that the duplication process shows little respect for gene boundaries and can spawn remarkably diverse sets of duplication products with varying degrees of structural resemblance to the ancestral copy. At one end of the spectrum, small-scale duplication (SSD henceforth) events faithfully duplicate the entire ancestral open reading frame (ORF) and possibly large stretches of upstream and downstream flanking regions, thereby capturing important ancestral *cis*-regulatory elements such as promoters. At the opposing end of the spectrum, other SSD events can display immense promiscuity by fashioning novel ORFs from both coding and noncoding genetic material ([21–24], among others). Furthermore, the recent discovery of the creation of *de novo* genes in entirety from noncoding DNA [25–31], although not duplicative in nature, completely turn Müller's [5] and Ohno's dictum [16] of “every gene from a pre-existing gene” on its head.

In this paper, I focus on the diversity of the gene duplication process whereby new genes are created by incorporating genetic tracts from previously existing genes as well as noncoding DNA (intergenic and intronic), and the evolutionary consequences of this promiscuity inherent in the gene duplication process. First, I describe the canonical model of gene duplicate evolution as envisioned by Ohno and delineate its major tenets as well as its failure to encapsulate the full complexity of the gene duplication process as revealed by whole-genome sequence data. Second, I discuss the various flavours of gene duplicates originating from both DNA- and RNA-mediated mutational events and explore their respective potential for the creation of evolutionary innovations and biological diversity. Third, I explore the various scenarios under which gene paralogs can escape homogenization by ectopic gene conversion, rendering them free to evolve along novel evolutionary trajectories and assume divergent functions. Lastly, I explore theoretical population-genetic considerations of how the effective population size (*N*_*e*_) of a species may influence the probability of emergence of genes with radically altered functions.

## 2. Ohno's Canonical Model of Gene Duplicate Evolution

**Figure 1:**
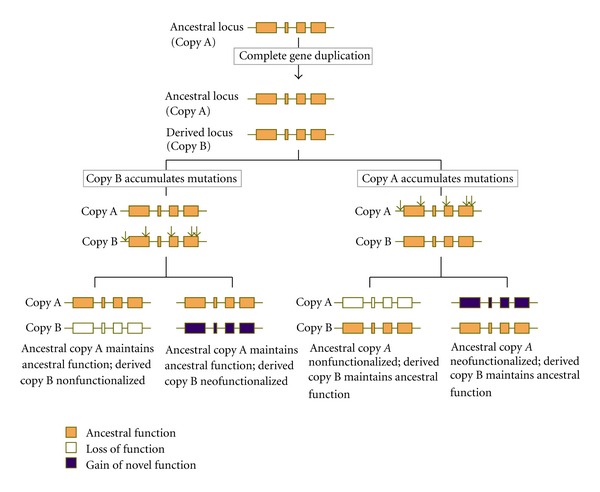
Different evolutionary fates of gene paralogs under Ohno's canonical model of gene duplicate evolution [16]. Ohno postulated that gene duplicates are born redundant in sequence and function to the ancestral copy. As such, either copy (ancestral or derived) has the potential to assume the identity of the locus maintaining the ancestral function leaving the other paralog free to accumulate mutations that may in turn engender vastly different evolutionary outcomes, namely loss of function (nonfunctionalization) or gain of a new function (neofunctionalization). A third evolutionary outcome not displayed in the schematic is the conservation of the ancestral function by both paralogs, in instances where natural selection favours increased gene dosage for increased levels of the ancestral gene product.

Ohno's overly restrictive view of the gene duplication process is related to his implicit assumption that the gene duplication process yields an extra copy that is fully redundant to the ancestral copy, both at the functional and sequence level. For this requirement to hold, the entire ancestral repertoire of coding sequence and regulatory elements would have had to be replicated during the duplication process. This supposed complete redundancy between duplicate copies then necessitates the implicit prediction that either copy, the ancestral or the derived, is capable of assuming (i) the ancestral function or (ii) becoming neofunctionalized/nonfunctionalized (see Figure 1). As such, the evolutionary fate of a duplicate copy under Ohno's model then rests on chance or stochastic events; the first gene copy to be hit by mutations, be they degenerating or neofunctionalizing, will be more prone to an altered evolutionary fate. We now know that gene duplicates, especially those stemming from SSD events often do not meet this assumption of functional equivalency to the ancestral copy at birth. In Ohno's defense, experimentally determined sequence data in the 1960s for newly originated gene duplicates from SSD events was scant at best, in direct contrast to the more abundant chromosomal complement data. For example, the high diploid chromosome numbers in modern vertebrate lineages and the vast differences in their chromosomal complements led Ohno and his colleagues to conclude that gene duplication by polyploidization constituted an “obligatory evolutionary requirement” for the evolutionary diversification of vertebrates [32–36]. It appears that Ohno himself was acutely aware that his model of gene duplicate evolution could possibly fall short of encapsulating the full complexity of the process, given the paucity of experimental data in his time. In the Introduction section of his 1970 treatise, he acknowledges: “In this golden age of biology, a book faces the danger of becoming obsolete before its publication. It is my belief that in order to avoid early obsolescence, the author, judging on the basis of the scant evidence available, is obliged to anticipate future developments and paint a picture with broad strokes of his brush. This I have done rather freely in this book.”

Ohno's model of functional equivalency of gene duplicates at birth was likely influenced by his views on the seminal role of polyploidization in evolution. Whole-genome duplication (WGD) or polyploidization offers several inherent advantages over SSD events such as tandem gene duplications, each of which were discussed in detail by Ohno [16]. First, gene duplication by polyploidization does not disrupt ancestral gene dosage ratios between functionally related genes. Second, the simultaneous duplication of all genes within an ancestral genome “in one fell swoop” via polyploidization would appear to have far greater evolutionary potential for functional diversification than SSD events creating one or a few duplicate copies at a time. However, rapid advancement of molecular techniques that enable genome-wide analyses of DNA content in conjunction with the use of experimental lines maintained under strict bottlenecking conditions to permit the accumulation of mutations under relaxed selective constraints have now provided evidence for astoundingly high genome-wide rates of spontaneous gene duplication via SSD events in the yeast, *Saccharomyces cerevisiae *[37] and the nematode, *Caenorhabditis elegans* [38] that exceed the base substitution rate by several orders of magnitude. These high per-locus duplication rates directly contribute to the immense copy-number variation being observed in various species [39–55]. Third, polyploidization also entails the coordinated duplication of the structural gene and associated *cis*- and *trans*-regulatory elements, thereby reducing the frequency of gene duplicates that are already nonfunctionalized at inception (“dead at birth”) owing to the incomplete duplication of their regulatory systems. These advantageous characteristics of WGD-originated gene duplicates likely influenced Ohno's views on what comprised the most evolutionarily successful class of gene duplicates stemming from SSD events—essentially, *complete* gene duplication events where the ancestral coding sequence and the entire ancestral repertoire of regulatory elements were inherited intact in the derived paralog. In other words, Ohno's canonical model of gene duplicate evolution only focused on one particular class of gene duplicates arising from SSD events (*complete* gene duplicates) that, at inception, most resembled paralogs derived from polyploidization events. It is not that *partial *gene duplicates produced by incomplete duplication events were unknown to science; in fact, commencing in the early 1960s, a handful of studies had already established their existence [13–15] and discussed the implications of the creation of such genes with drastically altered reading frames for the origin of evolutionary novelties [13]. It is not clear why Ohno overlooked entire classes of SSD duplicates derived from either incomplete duplication of a (i) single locus or (ii) multiple loci when arriving at his model of functional diversification of gene duplicates. Perhaps he believed that the majority of these duplicates were likely rendered nonfunctionalized at birth and as such, were rapidly eliminated from the population with minor or no evolutionary potential.

## 3. DNA-Mediated Duplication Events

### 3.1. Mechanisms of Mutation

DNA-mediated duplication (and deletion) events can originate via three mechanisms, namely (i) nonallelic homologous recombination (NAHR), (ii) nonhomologous end joining (NHEJ) and (iii) replication slippage.

Non-allelic homologous recombination (NAHR henceforth), also known as ectopic homologous recombination, refers to meiotic recombination between nonallelic but highly similar paralogous tracts of DNA that are already present in the genome. These extant paralogs in the genome are also referred to as low-copy repeats (LCRs) in the medical genetics literature [56, 57]. The NAHR pathway is by far the most precise means to repair double-strand breaks with no or minor loss of genetic information because it replicates the missing information from one homologous chromosome to another (interchromosomal) or between sister chromatids (interchromatid) or within the same chromatid (intrachromatid) [58, 59]. NAHR amongst paralogs in direct transcriptional orientation simultaneously leads to a duplication and deletion product each whereas NAHR among inverted paralogs results in inversions [59]. Unequal crossing-over events contributing to duplications are but NAHR events between paralogs in genomic proximity [60]. NAHR also requires extensive DNA sequence identity between paralogs in order to proceed (reviewed in [57]), approximately 50 bp in *E. coli* [61] and up to 300 bp in mammals [62, 63]. In contrast to NHEJ, which is facilitated by small DNA tracts of microhomology or no homology, paralogous DNA segments (or LCRs) facilitating NAHR are deemed lengthier with 95–97% sequence identity. Stankiewicz and Lupski [56] initially defined LCRs as ranging from 10–400 kb in size but a more recent paper by Hastings and colleagues has refined the criteria to encompass any paralogous segments >1 kb in size and with >95% sequence identity [57]. NAHR serves as a major contributor to genomic rearrangements such as duplications and deletions; Kidd et al. [64] inferred NAHR as having the greatest contribution to the formation of duplicates (~42%; 41/98 events) and 38% (49/129 events) of all deletion events in their analysis of structural variants in eight human genomes.

 Nonhomologous end joining (NHEJ henceforth) is another important contributor of duplications and deletions and like NAHR, is a recombination repair pathway for double-strand breaks in multicellular eukaryotes [65]. However, NHEJ differs from NAHR in that it requires little or no sequence homology. Hence, the NHEJ pathway is often described as being homology-independent. NHEJ works to modify the two broken ends of a double-strand break, rendering them compatible and capable of rejoining but with concomitant loss of genetic information; as such it is a far more imprecise repair mechanism. The fact that it is commonly employed for DNA repair in multicellular eukaryotes despite its imprecise nature has been somewhat of a puzzle. Lieber et al. [58] have proposed that NHEJ is far more efficient in effecting DNA repairs in highly repetitive regions of the genome compared to the NAHR pathway, hence its common deployment in multicellular eukaryotes whose genomes comprise a substantial fraction of repetitive DNA elements. While NAHR is restricted to late S or G2 of the cell cycle, the NHEJ pathway is ubiquitous and can function through all phases of the cell cycle [65]. NHEJ events can lead to complex structural changes simultaneously involving duplications, deletions and inversions with microhomology junctions ([66, 67]; reviewed in [57]) and were found to contribute to the origin of ~30% and ~45% of the characterized duplications and deletions, respectively, in the human genome [64].

Slipped-strand mispairing or replication slippage is a third mechanism mediating duplications and deletions of DNA fragments. By the 1980s, multiple independent studies had already reported on the existence of minisatellites or VNTRs and the hypervariable genetic variation associated with their occurrence [68–70]. This was rapidly followed by the discovery of microsatellites or short tandem repeats (STRs) or simple sequence repeats (SSRs) [71]. Replication slippage or slipped-strand mispairing can lead to both duplications and deletions of genomic regions associated with these STRs [72–74]. The proximity of the repeat sequences and their high degree of sequence homology are expected to have a saltatory effect on gene family expansion and shrinkage [75].

### 3.2. Complete Gene Duplicates

**Figure 2:**
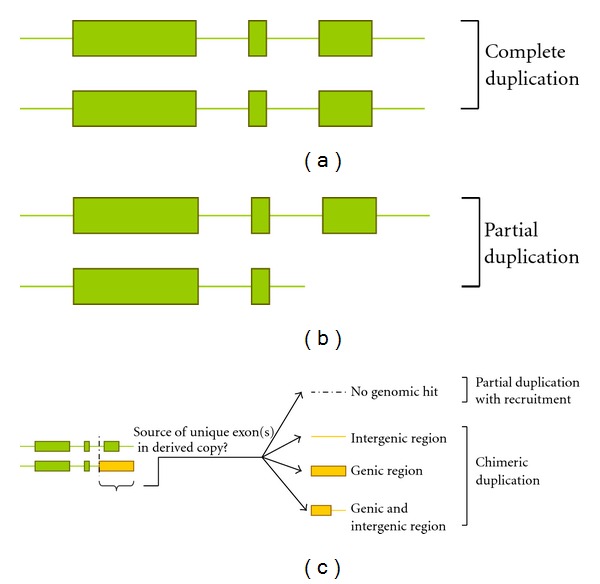
Small-scale DNA-mediated duplication events can yield daughter loci with varying degrees of structural resemblance to the ancestral copy depending on the extent of the duplication span and the location of the duplication breakpoints. Rectangles represent exons and solid horizontal lines through exons denote introns and flanking region sequences. Corresponding colours between the ancestral (top) and the derived (bottom) locus denote sequence homology. (a) *Complete* gene duplication wherein the duplication event spans, at a minimum, the entire ORF of the ancestral locus from the initiation codon to the termination codon. The duplication event may or may not encompass upstream and downstream flanking region sequences. In the schematic, *complete* duplication of an ancestral locus yields a derived copy (bottom) comprising three exons and intervening introns as well as some 5′ and 3′ flanking region sequences. (b) *Partial* gene duplication wherein only a portion of the ancestral ORF is duplicated. In the schematic, the downstream duplication breakpoint occurred within intron 2 of the ancestral copy (top), yielding a truncated derived copy (bottom) comprising some of the ancestral 5′ flanking region sequence, exons 1 and 2 and part of intron 2. (c) *Chimeric* gene duplication or *partial* gene duplication with *recruitment*. In instances wherein the derived locus (bottom) has unique exon(s) to the exclusion of the ancestral copy, the type of duplication event depends on the genomic source(s) of the unique coding region(s). In the schematic, the derived copy (bottom) has a unique exon 3 (yellow) to the exclusion of the ancestral locus (top). If a BLAST query of the unique exonic sequence yields no hits in the genome, it is suggestive of a *partial *gene duplication event and subsequent recruitment of intergenic sequence by the duplicate copy from its new genomic neighbourhood to yield an intact ORF. To qualify as a *chimeric* duplicate, this unique coding region of the derived locus must also exhibit evidence of duplication from another genomic source, be it intergenic, genic or a combination of genic and intergenic regions. The creation of such fusion genes could have occurred as a single evolutionary event (a single duplication event encompassing portions of two adjacent genes) or may represent independent duplication events and subsequent fusion of these fragments via shuffling.

*Complete* gene duplications are characterized by the duplication of an entire gene (Figure 2(a)). A strict adherence to Ohno's model of gene duplication then necessitates that for a *complete* duplicate to be redundant in both sequence and function to the ancestral copy, the entire ancestral coding region and regulatory elements would have to be inherited by the duplicate copy. Because *cis*-regulatory elements are poorly annotated in most genomes, efforts aimed at *complete* duplicate identification have relied entirely on a direct comparison of the ORF nucleotide sequences of the two paralogs. The paralogs exhibiting nucleotide sequence homology between their initiation and termination codons (including introns, when present) have traditionally been classified as *complete* duplicates. Therefore, some unknown proportion of the *complete* gene duplications identified in the manner described above likely had the ancestral ORF sequence duplicated without the concomitant duplication of the ancestral repertoire of regulatory elements, which may induce a divergent evolutionary trajectory for the newly created paralog at conception itself. As such, a subset of putative *complete* duplicates would fail to meet Ohno's strict definition of derived copies being created functionally identical to the ancestral locus. The actual proportion of *complete* duplicates that display functional divergence from the ancestral locus at or close to birth despite inheriting the ancestral copy's full coding sequence remain to be characterized via detailed functional assays.

#### 3.2.1. Contrasting Proportions of Complete Duplicates between Unicellular and Multicellular Eukaryotes

Only a handful of studies have identified the relative frequencies of *complete* duplicates relative to other structural categories of duplicates (*partial *and *chimeric*) in an entire genome or a particular age-cohort of duplicates within a genome (Table 1). For reasons identified in the preceding paragraph, the number of true *complete *duplicates bearing complete functional redundancy to the ancestral locus may be significantly fewer in frequency than reported in these studies. A study of putative evolutionarily young gene duplicates (synonymous divergence *dS* < 0.1) comprising small multigene families in the *Caenorhabditis elegans* genome found that *complete* duplicates comprised only ~40% (114/290) of all duplicate pairs considered [76]. *Drosophila* genomes tend to have remarkably similar proportions of *complete* duplicates to *C. elegans* within their cohort of evolutionarily young gene duplicates; ~41% in *Drosophila melanogaster* [24] and ~44% in *D. pseudoobscura* [77]. Contrastingly, the proportion of extant *complete* duplicates stemming from SSD events in the sequenced *Saccharomyces cerevisiae* genome is significantly greater at 82% (18/22 pairs) [78].

The contrasting proportion of *complete* duplicates in multicellular eukaryotes (*C. elegans*, *D. melanogaster*, and *D. pseudoobscura*) relative to the unicellular eukaryote *S. cerevisiae* begs further evaluation. Clearly, more genomes within diverse taxonomic groups and across kingdoms will have to be evaluated to generate robust sample sizes and greater phylogenetic independence in order to enable strong generalizations. However, if prokaryotes and unicellular eukaryotes are indeed verified as having a majority of *complete *duplicates (among genes of SSD-origin only) and multicellular eukaryotes a minority, there are several scenarios that may, individually or in concert, contribute to this pattern. First, assuming that DNA-replication errors and double-strand breaks are likely to yield similar distributions of duplication spans across diverse genomes, the probability of encompassing an entire gene during duplication is greater in compact genomes with smaller average gene lengths (shorter exon length, fewer introns, and/or shorter intron length). Conversely, in species with lengthier average gene lengths due to the elongation of genic coding regions and/or the presence of numerous lengthy introns, gene duplication is less likely to capture an ORF in its entirety, yielding a greater number of partial gene fragments. The average length of coding sequences in eukaryotes was found to be 445 bp longer than their prokaryotic counterparts [79]. Coding sequence differences may therefore contribute marginally to average gene length differences between the two kingdoms. However, average intron lengths and intron densities should have a far greater contribution to variation in average gene length between lineages given that (i) they can be highly variable across diverse taxa [80–85] as well as between closely-related taxa [86] and (ii) duplication across introns is part and parcel of DNA-mediated duplication events. The median gene lengths within the *S. cerevisiae* and *C. elegans* genomes do not appear starkly different (1.1 and 1.4 kb, resp.) and yet the frequencies of *complete* duplicates in their genomes are substantially so (82% and 40% in *S. cerevisiae* and *C. elegans, *resp.) [78]. This suggests that some other factor(s) may be implicated in the observed differences in the frequencies of extant *complete* duplicates within their genomes.

A second possibility is that structurally heterogeneous duplicates such as *partial* and *chimeric* duplicates may have deleterious effects at birth and may be more effectively eliminated by purifying natural selection in some genomes relative to others, given that the efficacy of natural selection is greater in genomes of species with larger effective population sizes (*N*_*e*_). This concept is discussed in greater detail in Section 6 below. The first sequenced genomes to become available were often of individual(s) derived from laboratory strains or natural populations. As such, the source population of the sequenced individual(s) may have already been subject to selection (or genetic drift) and characterization of all current duplications would not represent the entire spectrum of spontaneous duplications that may have arisen within the genome in the recent past. In other words, some duplications with deleterious effects may have already been purged from the genome in their infancy prior to their identification in whole-genome sequence data, leading to underestimates of the spontaneous duplication rate as well as a skewed pool of gene duplicates with lower rates of loss. Extending this reasoning to the observed frequencies of *complete* duplicates in various sequenced genomes, it is not possible to distinguish whether the high frequency of extant *complete *duplicates, say in the initial whole-genome sequence of *S. cerevisiae* [87], is due to a higher spontaneous rate of *complete *gene duplication or greater purifying selection acting to weed out *partial* and *chimeric* duplicates. The spontaneous rate of gene duplication and the spectrum of different classes of gene duplications have been characterized in long-term *C. elegans* mutation accumulation lines with severely diminished efficacy of natural selection [38]. Lipinski et al. [38] characterized 30 duplicated genes via DNA-mediated duplication events, of which ~37% (11/30) were *complete* duplicates which is in accord with the observed frequency of 40% *complete* duplicates in the originally sequenced genome of the *C. elegans* N2 laboratory strain [76]. The picture is far from clear for genomes of prokaryotes and unicellular eukaryotes with large *N*_*e*_. The initially sequenced genome of *S. cerevisiae* has relatively few extant gene duplicates created from SSD-events [78, 88]. While this may initially give the appearance of a low spontaneous rate of gene duplication, whole-genome sequencing of yeast mutation accumulation lines has revealed, to the contrary, an extremely elevated rate of spontaneous duplication and deletion far exceeding the base substitution rate [37]. This suggests that most duplication/deletion events in yeast are selectively purged from the genome in their infancy. The relative frequencies of spontaneously arising *complete*, *partial*, and *chimeric* duplicates in yeast has not yet been experimentally determined. It may be possible in the future to conclude whether the higher frequency of *complete* duplicates in the initially sequenced *S. cerevisiae* genome is owing to (i) a higher rate of *complete* gene duplication, or (ii) a greater efficacy of selection against structurally heterogeneous gene duplicates (*partial *and *chimeric*), or (iii) a combination of scenarios (i) and (ii).

#### 3.2.2. Are Complete Duplicates More Limited in their Ability to Explore Evolutionary Space and Assume Radically Novel Functions?

*Complete *duplicates as defined under Ohno's classical model of gene duplicate evolution commence their evolutionary life structurally and functionally redundant to the ancestral copy from which they are derived. If an altered environmental regime induces a selective pressure for amplification of an ancestral gene product, *complete* duplicates are the best poised relative to other structural classes of duplicates (such as *partial *and *chimeric *duplicates) to assist the ancestral copy in responding to cellular needs for “more of the same.” Gene amplification involving segmental duplications of gene clusters yielding mostly *complete* duplicates is known to enable bacterial growth in carbon-limited environments [89–91] and the evolution of antibiotic resistance [92]. A similar pattern is observed in multicellular eukaryotes. Gene amplification is implicated in copper resistance by yeast via tandemly arrayed duplications of the *CUP1* locus [93], insecticide resistance [94, 95], and heavy metal tolerance by insects [96], in the recruitment of lysozyme as a major stomach enzyme in cows [97] and in protozoan resistance to drugs [98]. The initial preservation of the duplicate copy under selection for increased dosage does not preclude eventual functional diversification of the two paralogs via refinement of their secondary functions as envisioned under the IAD (innovation, amplification, divergence) model of Bergthorsson et al. [99].

Ohno [16] believed that the extra copy first accumulates debilitating “forbidden” mutations leading to a loss of function, which in turn instigates its evolution along an altered evolutionary trajectory under a regime of relaxed selective constraints. While the majority of the accumulating mutations in the extra copy are expected to be nonfunctionalizing, a rare beneficial mutation may arise and impart a novel function, thereby facilitating its resurrection. This has also been referred to as the *mutation during nonfunctionality* (MDN) model [100]. If sequence and functional divergence between paralogs is largely a consequence of point mutations in the postduplication period, how might this influence the evolutionary potential of *complete *duplicates? There are instances of *complete* duplicates whose evolution proceeds along the trajectory envisioned by Ohno, such as visual pigment proteins in catarrhine primates [101] and pancreatic ribonuclease paralogs in colobine primates [102]. Catarrhine primates (Old World monkeys, apes, and humans) have trichromatic vision due to an evolutionarily recent X-linked duplication yielding the red and green opsins, each of which comprises six homologous exons in humans, pygmy chimpanzee, gorilla and orangutan [103]. Hence, red and green opsins represent a *complete* gene duplication event. The encoded opsin proteins differ intraspecifically by 12–18 amino acids [103], of which as few as three residues (positions 180, 230 and 285) have been shown to account for the spectral difference of *≈*30 nm in peak absorption values between the red (L opsin) and green (M opsin) photopigments [104–106]. Interestingly, one species of Platyrrhine monkeys, the howler monkey, displays convergent evolution via duplication of the X-linked L opsin to yield a new M opsin independently of the Catarrhine primates ([107] reviewed in [108]). In both instances, acquisition of a novel function, the evolution of a novel green photoreceptor encoded by the M opsin via duplication of the L opsin (encoding the red photoreceptor) was effected by the accumulation of point mutations in the coding regions. Alternatively, the accumulation of point mutations in regulatory regions of a *complete* duplicate has the potential to alter tissue specificity or temporal expression patterns relative to the ancestral copy. However, I propose that, collectively speaking, *complete* duplicates have markedly less potential to assume “radically” novel functions from their progenitor copy. The argument that the creation of structurally heterogeneous duplicates with a radical refashioning of ancestral exon-intron structure can lead to immediate acquisition of drastically novel function conferring a great selective advantage has been presented before [13, 23, 75]. A gradual increase in sequence divergence between *complete* duplicates via point mutations can lead to minor tinkering of the ancestral function or an alteration of expression patterns. Successive mutational hits by base substitutions in one paralog could lead to incremental changes in its function but it might require substantial evolutionary time to evolve a drastically altered novel function if other alterations to exon-intron structure via indels or shuffling events are absent. Despite 35–40 million years of evolutionary divergence from its progenitor, the green opsin gene of Old World primates is still very much a photoreceptor gene as is its ancestral paralog. The origin of radically altered functions might be more the domain of *partial *and *chimeric *duplicates with extensive changes to their exon-intron structure.

### 3.3. Partial Gene Duplicates

*Partial* gene duplications are also referred to as incomplete, intragenic, or internal gene duplications and are characterized by the incomplete duplication of an ancestral gene (Figure 2(b)). In some instances, *partial* gene duplication is additionally associated with *de novo* internal amplification of short DNA tracts. The most accurate means of identifying *partial* duplicates would be to align the ORFs of a known ancestral and derived copy within a duplicate pair and visually determine if the derived copy is a truncated version of the ancestral ORF sequence. In some instances, the derived copy's ORF may appear superficially attenuated in length relative to that of the ancestral copy but its 5′ and/or 3′ flanking sequence will exhibit complete sequence homology to the ancestral ORF. These cases should be treated as *complete* duplication events because they are suggestive of a role of postduplication events in the alteration of the derived copy's ORF. For example, base substitutions or frameshift mutations can lead to the conversion of an ancestral sense codon to a premature stop codon in the derived copy's ORF. Conversely, the derived paralog's ORF may appear superficially lengthier than its ancestral counterpart, but sequence analysis may reveal a true *partial* duplication associated with massive internal gene amplification of a small ancestral DNA sequence tract. A further complicating factor in the accurate identification of *partial* duplicates may be posed by the presence of unique coding sequence in the derived copy's ORF, to the exclusion of the ancestral copy [23]. This may lead to the erroneous conclusion that the derived copy is a *chimeric* duplicate derived from the duplication of multiple genomic sources. However, if the unique sequence of the derived copy fails to generate any valid genomic hits for potential donor sequence(s) via a BLAST search, it is suggestive of a *partial duplication with recruitment* (see top panel of Figure 2(c)). In other words, the derived paralog was formed via a *partial* duplication of the ancestral copy and additionally recruited neighbourhood sequence from its new genomic location to complete its ORF. For reasons outlined above, accurate identification of structurally heterogeneous classes of duplicates such as *partials* and *chimerics* are more challenging than *complete* duplication events.

 Determining the ancestral and derived copy within duplicate pairs showing *partial *structural resemblance is best facilitated by comparing the exon-intron structure of the two paralogs within the focal genome to that of a single-copy ortholog in a closely-related outgroup genome. The paralog within the focal genome displaying greater similarity in exon-intron structure to the single-copy ortholog is taken to represent the ancestral paralog. However, in the absence of outgroup genomic sequences, the earliest studies tentatively classified paralogs as *partial* duplicates when direct examination of their ORF sequences revealed that one paralog had unique coding sequence to the exclusion of the other paralog (e.g., [23]). That is, the shorter paralog had a truncated ORF and displayed no sequence homology with the lengthier paralog beyond the putative duplication breakpoint within its ORF. The disadvantage of this approach to *partial* duplicate identification is the possibility that the shorter copy is actually the ancestral paralog and the lengthier copy may have resulted from a *complete* gene duplication of the ancestral ORF and the addition of extra sequence via recruitment of noncoding DNA or shuffling events involving other genic regions. In a subsequent study, Katju and Lynch [23] utilized the *C. briggsae* genome as an outgroup to assign ancestral versus derived copy status to paralogs within a subset of 14 *C. elegans* duplicate pairs that had been previously classified as* partial* duplicates [76]. The authors found that 13/14 (93%) of these were indeed *partial* duplications in that the lengthier *C. elegans* paralog was the ancestral copy given its exon-intron structure resembled that of the single-copy *C. briggsae* ortholog. In only one instance, the shorter paralog was the ancestral copy with the lengthier paralog representing a novel chimeric gene derived from multiple genic and intergenic sources. More interestingly, 43% of a subset of *C. elegans* 23 duplicate pairs previously identified as *chimeric* duplicates (both paralogs had unique coding sequence to the exclusion of the other copy) were actually cases of *partial duplication with recruitment* (see Figure 2(c)). As such, their original study [76] likely underestimated and overestimated the number of *partial* and *chimeric* duplicates, respectively.

 Given that a mere comparison of exon-intron structures of two paralogs may belie the actual type of duplication contributing to the creation of the extra copy, I propose a stricter definition of what comprises a true *partial* gene duplication event. Most crucially, an accurate assignment will require independent verification of the identities of the ancestral versus derived copy via comparative genomic approaches. A *partial* duplicate should only be derived from the partial duplication of a single ancestral genic source. In some instances, the *partial* duplicate may possess unique ORF sequence to the exclusion of the ancestral copy, leading to the erroneous conclusion that it is a *chimeric* duplicate. If this unique ORF sequence of the derived copy fails to generate any hits in the genome (exonic, intronic or intergenic), it should be classified as originating from a *partial duplication event with recruitment* wherein additional neighbouring sequence from the derived paralog's new insertion site were utilized to fashion novel exon(s) and/or intron(s) (see first panel in Figure 2(c)). Because these novel exon(s) and/or intron(s) in the duplicated copy are not derived from independent duplication events involving subsequent shuffling and fusion, as such the derived paralog is still very much a *partial* duplicate despite its superficial chimeric appearance. For example, the *Hun* gene in *Drosophila* is thought to have arisen from a *partial* duplication of the *Bällchen *gene with additional recruitment and exonization of flanking intergenic sequence [109]. Hence, *Hun *ought to be classified as a *partial duplicate with recruitment, *not a* chimeric *duplicate, as its novel ORF sequence (comprising ~33 amino acids) is not derived from an independent duplication event.

#### 3.3.1. High Genomic Abundance of Partial Duplicates in Multicellular Eukaryotes

Relatively high frequencies of *partial* duplicates have been identified in the sequenced genomes of *C. elegans*, *D. melanogaster*, *D. pseudoobscura*, and *S. cerevisiae* and directly falsify one of the major tenets of Ohno's model [16] that gene duplicates are created structurally redundant to their ancestral counterparts. Katju and Lynch's 2003 structural analysis of *C. elegans* paralogs [76] lacked a reference outgroup genome for comparison as the *C. briggsae* sequenced genome had yet to be released. *C. elegans* duplicate pairs with paralogs of differing amino acid lengths wherein the entire ORF of the shorter paralog was homologous to the lengthier paralog's ORF but the latter had unique ORF sequence to the exclusion of the shorter paralog were classified as putative *partial* duplicates. This class of *partial* duplicates comprised 21% of 290 duplicate pairs with synonymous divergence per synonymous site (*dS*) ranging from 0 to 0.1 (Table 1). As discussed in the previous section, this is likely an underestimate as a subsequent analysis by the authors using the *C. briggsae* genome as an outgroup showed that 43% of a subset of 23 *chimeric* duplicate pairs in reality represented *partial duplicates with recruitment *[23]. If the results generated from the subset of 37 *C. elegans* duplicate pairs can be generalized to the entire data set of 290 duplicate pairs, *partial* duplicates may represent ~38% of young *C. elegans* duplicates (rather than 21%) and *chimeric* duplicates only 23% (instead of 40%). These predictions await further experimental validation via a comparative genomic approach identifying the ancestral versus derived paralog for all duplicate pairs in *C. elegans*. Zhou et al. [24] reported *partial* duplicates as comprising 27% of newly originated genes in *D. melanogaster *(Table 1). Meisel's [77] study utilized a single-copy *D. melanogaster* ortholog as an outgroup to determine the directionality of structural alterations, if any, within *D. pseudoobscura* paralogs. This is a far superior approach as it enables accurate classification of the different structural classes of paralogs within a genome. The majority of *D. pseudoobscura *duplicate pairs (53%) were classified as *partial* duplicates (Table 1). The unicellular eukaryote, *S. cerevisiae*, has an extremely low percentage of identifiable *partial* duplicates, at about 7% (Table 1) even though older cohorts of gene duplicates with *dS* ≤ 0.35 were included in the analyses [78]. Furthermore, some fraction of these yeast duplicates may be evolutionarily older than that suggested by their *dS* values given the high degree of paralog homogenization in this genome due to the concerted action of codon usage bias selection and ectopic gene conversion. As discussed in Section 3.2.1, the paucity of structurally heterogeneous gene duplicates (*partial* and *chimeric*) in *S. cerevisiae* may be due to (i) a higher probability of *complete* duplicate origin given the compact nature of the genome and/or (ii) stronger purifying selection against structurally heterogeneous paralogs.

Two studies, one of experimentally evolved laboratory lines and the other of natural isolates report the highest fraction of *partial* duplicates thus far (Table 1). Lipinski et al.'s [38] analysis of long-term *C. elegans* mutation accumulation lines found *partial* duplicates accounting for 63% of detectable spontaneous gene duplication events. As with the Lipinski et al. study [38], Meisel [77] found zero frequency of *chimeric* duplicates in *D. pseudoobscura*. These results suggest the tantalizing hypothesis that while *chimeric* duplicates can be created in one fell swoop by duplication across two adjacent genes, the majority of them may owe their creation to secondary fusion events involving the recombination of previously duplicated *partial *duplicate fragments. A population-genomic study of evolutionarily young genes still segregating as copy-number variants (CNVs) in 15 *D. melanogaster* natural isofemale lines identified 76% of all duplications to be *partial *duplicates [45]. It is not clear if CNVs associated with these *partial *duplicates are indicative of insufficient evolutionary time for fixation or represent the incipient stages of eventual loss from the genome.

#### 3.3.2. Partial Duplicates Can Encode Drastically Novel Functions Relative to the Ancestral Copy

*Partial* duplicates were most certainly overlooked by Ohno as having much evolutionary potential. Even in the current genomic era, the common trend is to lump together *partial *duplicates as pseudogenes given that their ORFs show signatures of disruption to the ancestral reading frame and the presence of premature stop codons. While many *partial *duplicates may indeed be evolutionary dead ends, their high rates of origin and potential for evolution of radically novel functions due to their drastically altered exon-intron structure relative to the ancestral copy urges some measure of caution against, in common parlance, throwing the baby out with the bathwater. Furthermore, *partial* duplicates may remain nonfunctional in a genome for some evolutionary time, but may be resurrected by exapting to novel functions under altered environmental regimes. In the paragraph below, I highlight some intriguing examples of acquisition of radically altered function by *partial *duplicates (listed in Table 2).

Freezing avoidance by various polar and subpolar species of fishes highlight the remarkable nature of adaptation in living organisms owing to the presence of certain antifreeze proteins that evolved independently from functionally unrelated ancestral genes. The molecular origins of these antifreeze proteins are testament to the evolutionary potential of *partial duplication *events in conjunction with *de novo* internal amplification of short sequences. The antifreeze glycoprotein (AFGP) of Antarctic notothenioid fish was likely created by a *partial duplication *of an ancestral pancreatic enzyme trypsinogen wherein a small portion of its 5′ untranslated region, E1, I1, a small fragment of E2, terminal E6, and a portion of the 3′ untranslated region were initially duplicated. This was likely followed by the internal amplification of a 9 bp sequence straddling the first intron-second exon that eventually encoded the repetitive tripeptide backbone of the AFGP which directly contributes to the novel protein's ice-binding capacity and inhibitory effect on the growth of ice crystals [21]. Deng et al. [110] recently elucidated the independent evolutionary origin of another class of antifreeze proteins, the AFPIII (Type III antifreeze protein) in an Antarctic zoarcid fish from an ancestral sialic acid synthase (SAS) gene unrelated to trypsinogen. The two-exon AFPIII gene was derived from a *partial* duplication of the ancestral SAS gene, encompassing a portion of both its 5′ flanking region sequence and E1, I5, terminal E6, and portion of the 3′ flanking region sequence [110]. The AFPIII locus comprises >30 AFPIII genes arrayed in ~8 kb repeats with one AFPIII gene per repeat [110, 111]. E2 of AFPIII imparts the antifreeze property of the molecule and is derived wholly from the ancestral SAS terminal E6 exon. E1 of AFPIII encodes for a signal peptide for extracellular export of the mature antifreeze protein and was created *de novo* by combining 54 nt of 5′ flanking region upstream from the translation start site and inclusion of the first six codons of the ancestral E1 of SAS. Indeed, exonization of ancestral noncoding sequence, as is implicated in the origin of the first exon of the novel AFPIII gene, may have the greatest contribution to new domain gains in animal proteins relative to retrotransposition and recombination-mediated intronic insertion events [112].

*Partial duplications with recruitment* bear immense potential to generate radically novel functions due to exonization of ancestral noncoding sequences leading to the possible emergence of novel protein domains. Species in the genus *Caenorhabditis* employ one of two modes of reproduction. Nine of the first 11 species to be cultured display a gonochoristic obligate female/male outcrossing mode of reproduction. Two species, *C. elegans* and *C. briggsae,* have an androdioecious breeding system with populations composed of self-fertile hermaphrodites and males at a low frequency (<0.1%) [119]. Two independent lines of evidence suggest convergent evolution of hermaphroditism within *C. elegans* and *C. briggsae* as follows: (i) these two hermaphroditic species are phylogenetically separated by two gonochoristic species [120] and (ii) the sperm production pathway in the hermaphrodites of these two species involves different genes [121]. The evolution of hermaphroditism in *C. elegans* may have been specifically promoted by the appearance of a novel gene, *fog-2*, via a *partial* gene duplication of *ftr-1*, a gene of unknown function [113]. The appearance of *fog-2 *conferred *C. elegans* hermaphrodites with a limited ability to perform spermatogenesis [122, 123]. The ancestral locus, *ftr-1* comprises four exons encoding 314 amino acids (aa). The exon-intron structure of *fog-2*, comprising five exons (327 aa) exhibits both similarities and dissimilarities relative to *ftr-1*. *fog-2* was created by the duplication of *ftr- *1′s E1-E3 and part of E4. The C-terminal region of *fog-2* encompassing the latter half of E4, I5 and E5, as well as its 3′ flanking region bear no obvious sequence homology to *ftr-1*, nor do they generate any sequence hits in the *C. elegans* genome. Hence, *fog-2* was created by a *partial duplication with recruitment* event involving exonization of noncoding sequence from its new genomic neighbourhood to complete its open reading frame [113, 124]. Notably, the recruitment and subsequent exonization of this unique noncoding sequence in *fog-*2′s 3′ end may have facilitated neofunctionalization after duplication, given that this novel region is implicated in binding with a translation repressor GLD-1 that represses feminization and promotes hermaphrodite spermatogenesis [122]. The X-linked *Hun* gene in three *Drosophila* species represents another example of a sex-specific gene created by *partial duplication with recruitment* [109]. *Hun* was created by a *partial *duplication of the autosomal gene *Bällchen*, a kinase involved in germ cell development, with subsequent recruitment of new neighbourhood sequence into its terminal exon, leading to a novel testis-specific expression.

 More recently, the origin of an extra *SRGAP2* gene in the lineage leading to modern humans via a *partial duplication* event is being credited with major evolutionary changes related to brain development and advancement of cognitive abilities in the human lineage and its divergence from primate relatives [114, 115]. The ancestral *SRGAP2* gene comprising 22 exons (encoding 1071 aa) underwent several independent duplication events in the human-lineage leading to the creation of *partial* duplicates *SRGAP2B*-*SRGAP2D* spanning the first nine ancestral exons [115]. One of the *partial* paralogs, *SRGAP2C, *encoding a truncated version of the ancestral SRGAP2 protein product comprising 458 aa residues as well as 7 unique residues at the carboxyl terminus, is thought to dimerize with the ancestral protein product leading to a dominant negative interaction essentially involving the knockout of the ancestral gene function and facilitating (i) rapid neuron migration and (ii) the development of greater spine extensions on neuronal surfaces which in turn are thought to facilitate greater connections between neurons [114].

*Partial* duplicates have also been implicated in the regulation of the ancestral paralogs from which they are derived. The putative *partial* duplicate of the nitric oxide synthase (NOS) expressed in the central nervous system of the snail *Lymnaea stagnalis* appears to function as a translational regulator to inhibit the translation of the ancestral neuronal NOS protein [125]. The mouse *Makorin1-p1* is a truncated version of the *Makorin-1* gene and presumably arose via a *partial *gene duplication spanning 700 bp of the 5′ region of the ancestral, 2600 bp long *Makorin-1* gene [126]. The authors proposed that the mRNA expression of the *partial* duplicate *Makorin1-p1* inhibited degradation of the ancestral locus *Makorin-*1′s mRNA, thereby enhancing and stabilizing the ancestral gene's expression. Furthermore, evolutionary analyses suggested that *Makorin1-p1 *was not evolving under relaxed selective constraints as would be expected of a neutral locus [127]. However, the authenticity of Hirotsune et al.'s [126] conclusions were subsequently debated by Gray et al. [128] who argued that* Makorin1-p1* is not transcribed and the mRNA attributed to it was an alternatively spliced variant of the ancestral *Makorin-1* locus. Another intriguing example of a *partial* duplicate acting in a regulatory role is the *DM-W* gene in the African clawed frog, *Xenopus laevis*. *DM-W* functions in female sex-determination and appears to have originated from the *partial* duplication of the first four exons of the male-specific, six-exon autosomal gene *DMRT*1**β** [116, 117]. *DM-W* initiates primary ovary formation in female gonads by antagonizing the activation of male-specific genes by *DMRT1* via transcriptional repression [116, 118]. Likewise, targeted knockdown of *ABCC6P1*, a putative *partial* duplicate of the human ACB transporter genes *ABCC6* leads to a reduction in the mRNA expression of *ABCC6* [129]. Finally, the role of *partial *duplicates in the formation of small noncoding RNAs with novel regulatory functions has barely been touched upon [130]. For example, Guo et al. [131] identified 22,956 DNA-mediated “pseudogenes” in the rice genome with a subset of them being strong candidates for assuming novel regulatory functions as small RNAs. However, the exact structural nature of these putative “pseudogenized” paralogs has yet to be elucidated in detail.

### 3.4. Chimeric Gene Duplicates

Current literature is replete with examples of putative *chimeric *duplicates. The diversity of mechanisms that can lead to the formation of novel genes exhibiting a mosaic or chimeric appearance certainly adds to the confusion that abounds with respect to their classification. As discussed by Cardoso-Moreira and Long [132], a multitude of genomic rearrangements following gene duplication (such as deletions, inversions, and translocations) can lead to a chimeric gene structure. The difference between a chimeric appearing gene and a true *chimeric* duplicate is subtle but cannot be relegated to pure semantics, and hence ought not to be ignored. In their review article [132], Cardoso-Moreira and Long offer the following definition: “A new gene is considered chimeric if it recruits novel sequence from nearby regions.” But if its creation involved gene duplication, what class of gene duplicate would it represent? As I have highlighted in the *fog-2* example in Section 3.3.2, a *partial duplication with recruitment* event created the chimeric/mosaic structure of *fog-2*. A partial fragment of the ancestral *ftr-1* gene's ORF was duplicated in conjunction with exonization of new neighbourhood noncoding sequence to render an intact ORF. Because *fog-2* is derived from the duplication of only one ancestral source, it qualifies as a *partial* duplicate, albeit with *recruitment*.

 I propose that *chimeric* gene duplicates be classified as paralogs derived from the duplication of two or more ancestral donor sequences, with at least one donor sequence required to be of genic origin (hence the classification as a “gene” duplicate). This definition can accommodate a variety of DNA-mediated mutational events leading to the formation of *chimeric *duplicates. A single duplication event partially encompassing two adjacent genes can instantaneously create a *chimeric* duplicate derived from the juxtaposition of two partial ancestral gene fragments. This ought to be a common mechanism of* chimeric* duplicate creation, as the gene duplication process appears to have little respect for gene boundaries [76, 133]. *Chimeric* duplicates can also be created via shuffling events that fuse together partially duplicated fragments of disparate ancestral origins (exonic, intronic and intergenic). Of course, to qualify as a *chimeric* gene duplicate, at least one of the ancestral donor sequences would have to be derived from a genic source. Figure 2(c) graphically represents the various types of *chimeric* duplicates derived from DNA-mediated duplication events. Figure 2(c) displays two duplicate copies (upper and lower gene copies are the ancestral and derived paralog, resp.) with sequence homology across exons 1 and 2 and terminating in intron 2 (shaded in green). The lower derived copy has a unique nonhomologous, terminal exon 3 (shaded in yellow). While the derived copy exhibits a superficial chimeric gene structure, a final classification is dependent on whether or not the unique terminal exon is derived from a duplication event. If the unique exonic sequence of the derived copy fails to generate any valid Blast hits in the genome (i.e., fails to identify a potential ancestral donor sequence), the duplicate should be classified as a *partial duplicate with recruitment* (top panel of Figure 2(c)). Alternatively, any significant hits to (i) intergenic, (ii) genic (exonic and/or intronic), or (iii) combination of intergenic and genic sequences in the genome would constitute evidence for its classification as a *chimeric* duplicate (lower three panels of Figure 2(c)).

#### 3.4.1. Abundance of Chimeric Gene Duplicates in Genomes of Multicellular Eukaryotes

Among the three structural classes of gene duplication, *chimeric *gene duplicates are possibly the most challenging to classify accurately. *Partial duplicates with recruitment *superficially resemble *chimeric* duplicates and distinguishing between these two categories requires comparisons of exon-intron structure of both paralogs with a single-copy ortholog as well as additional investigations to further determine the existence of potential ancestral donor sequences for unique sequence tracts in the derived copy. In the absence of genome sequences of closely-related outgroup species to enable a comparative genomic approach to duplicate classification, early studies directly compared paralogous ORF sequences to indirectly estimate the frequency of *chimeric *duplicates. Katju and Lynch's study [76] of evolutionarily young *C. elegans *gene duplicates initially classified *chimeric* duplicates as comprising two paralogs of differing amino acid sequence length wherein sequence homology between the two copies was disrupted within the ORFs of both copies, such that both had unique ORF sequence to the exclusion of the other copy. However, this approach will fail to distinguish *partial duplicates with recruitment* from *chimeric duplicates*. Indeed, in a subsequent study using a comparative genomic approach, 43% of a subset of 23 *C. elegans* gene duplicates previously characterized as *chimeric* duplicates in the absence of an outgroup sequence were found to constitute *partial duplicates with recruitment* [23]. In their study of *Drosophila melanogaster *gene duplicates, Zhou et al. [24] classified a derived paralog as a *chimeric *duplicate if it possessed a >50 bp nonhomologous sequence to the exclusion of the ancestral copy. This approach too suffers from the inability to distinguish *partial duplicates with recruitment* from *chimeric duplicates*. As such, some unknown fraction of *D. melanogaster chimeric* duplicates as identified by Zhou et al. [24] likely represent *partial duplicates with recruitment*.

Table 1 reports the percentage of *chimeric* duplicates in four species. It is highly likely that measures of 40% (116/290 duplicate pairs) *chimeric* duplicates within *C. elegans* [76] and 32% within *D. melanogaster* [24] are overestimates due to the misclassification of *partial duplicates with recruitment *as *chimeric* duplicates. Emerson et al.'s [45] calculation of 10% *chimeric* duplicates in natural isolates of *D. melanogaster* are derived from direct observation of *partial* duplication events across two adjacent genes leading to the formation of *chimeric* duplicates, and as such represent a *bona fide* conservative estimate of the frequency of *chimeric* duplicates within these genomes. Irrespective, these measures of *chimeric* and *partial* duplicates taken together underscore the widespread existence of structurally heterogeneous duplicates within evolutionarily young cohorts of gene duplicates in multicellular eukaryotic genomes. More specifically, they directly contradict Ohno's assumption that gene duplicates commence their evolutionary life redundant in sequence and function to their ancestral counterparts.

As was the case with *partial* duplicates, *chimeric* duplicates in *S. cerevisiae* are observed in extremely low frequency (4%) [78]. Taken together, structurally heterogeneous duplicates only comprise 11% of yeast duplicates derived from small-scale, DNA-mediated duplication events. This is in direct contrast to the genomes of multicellular eukaryotic species like *C. elegans* and *D. melanogaster* wherein structurally heterogeneous duplicates (*partials* and *chimerics*) comprise 56–86% of all duplicates (Table 1).

#### 3.4.2. Evolutionary Potential of Chimeric Duplicates

A recognition of the evolutionary potential of chimeric genes is not new [134]. As is the case with *partial* duplicates, *chimeric* duplicates derived from the fusion of multiple duplicated frames of diverse genomic origins can play a significant role in the origin of evolutionary novelties. In *C. elegans*, *chimeric* duplicates were found to possess novel exons fashioned from diverse genomic sources including repetitive elements as well as exonic, intronic, and intergenic sequences [23]. Shuffling of fragments or domains can alter the regulation and functionality of the novel gene and facilitate its fixation at the species-level if the new function offers a selective advantage at the point of conception [135]. Because numerous examples of *chimeric* duplicates exist and have been extensively reviewed in preceding publications [132, 136, 137], for logistic purposes I restrict my discussion to a few cases.

 Several *chimeric* duplicates have originated from a single duplication event that partially overlapped two adjacent genes. Incomplete duplication across two ORFs would appear to entail a high probability of creating a degenerated novel ORF marked for a nonfunctionalizing fate. However, it appears that *chimeric* duplicates in *Drosophila*, a genus in which they are particularly well-studied, have appreciably lower rates of origin (~11 duplicates/my) relative to other DNA-mediated duplications (~80 duplicates/my) but are equally liable to be preserved in the genome [138]. It is also of considerable biological interest to elucidate if such *chimeric* duplicates created by the fusion of two ancestral genes are (i) equally divergent in function from either ancestral gene, or (ii) possess a function that resembles that of both ancestral genes, or (iii) disproportionately resemble one ancestral gene's function.

The* Sdic* cluster represents an interesting example of a *chimeric* duplicate in *D. melanogaster* [22]. Although derived from an incomplete duplication event across two independent ancestral genes, the mutational events altering its genomic organization subsequent to its formation resemble that of the *partial* duplicate AFPIII cluster [110]. The *Sdic *gene was created by an incomplete duplication event spanning the latter half of an upstream cytoplasmic dynein gene *Cdic *and the N-terminal region of its adjacent downstream neighbour *AnnX* which encodes for an annexin protein. Several internal deletions subsequent to the duplication event refashioned a novel ORF which was duplicated multiple times to form a tandem array of ~10 copies. Interestingly, *Sdic*'s function resembles that of its *Cdic* progenitor in that it too encodes for a dynein, except one whose expression profile has been substantially narrowed and altered to be testis-specific [22]. The *Qtzl *gene in *D. melanogaster* represents a recently derived *chimeric *duplicate created via incomplete duplication across adjacent ancestral genes *CG12264* and *escl* [139]. While the exact function of *Qtzl* remains to be ascertained, it exhibits a strong molecular signature of preservation by natural selection, namely a drastically reduced level of genetic diversity in its genomic location and rapid fixation across 35 natural isolates of *D. melanogaster* despite its recent evolutionary origin [139]. It is also interesting to note that the expression profile of *Qtzl *disproportionately resembles that of its *escl* parent despite the observation that the ancestral *escl* donor sequence was inherited out of frame. Therefore, it appears that both *Sdic *and* Qtzl* described above display (i) substantial narrowing of their spatial expression profiles and (ii) appear to have functional roles that disproportionately resemble one of the two parental genes contributing to their chimeric origin.

Opazo et al. [140] report on an intriguing example of a *chimeric *duplicate derived from the fusion of two ancestral globin genes that has proceeded to functionally supplant one of its parental genes. A proto *β*-globin gene duplicated in the common ancestor of eutherian mammals following divergence from marsupials to generate the adjacent paralogs HBB (*β*-globin) and HBD (*δ*-globin) [141–143]. Most eutherian lineages have seen the deletion or degeneration of the HBD paralog with functional haemoglobin products encoded for by one or more HBB genes [143]. Paenungulate mammals comprising the three orders of Proboscidea (elephants), Sirenia (dugongs and manatees), and Hyracoidea (hyraxes) possess a chimeric HBB/HBD (*β*/*δ*) globin gene derived from a duplication event across the HBB and HBD paralogs. The parental HBB and HBD genes have both been pseudogenized by a N-terminal deletion and 3.2 kb insertion, respectively. The *chimeri*c HBB/HBD duplicate in paenungulate mammals has assumed the functional role of its ancestral HBB gene and encodes for the *β*-chain subunits of adult haemoglobin.

## 4. RNA-Mediated Duplication Events

 Retrotransposition is another dominant mechanism facilitating the creation of gene duplicates. Such RNA-mediated duplication events, also referred to as retroduplications, occur when spliced messenger RNA of an ancestral locus is reverse transcribed into cDNA and then reinserted into a novel genomic position. Gene duplication by retrotransposition instantaneously creates a duplicate gene with diverged characteristics from its progenitor locus [144–146]. First, retroduplication typically creates a single-exon gene duplicate from a multiexonic ancestral gene. Second, because retroduplication only encompasses transcribed sequences, the duplicate copy inherently lacks the ancestral repertoire of regulatory elements that control the expression of its progenitor locus. Preservation of a functional retrocopy (often referred to as “retrogenes” or “processed genes”) is then dependent on the retrocopy's ability to fortuitously recruit a novel promoter and other key *cis*-regulatory elements. Third, retrocopies are randomly inserted into novel genomic locations and as such inherit a genomic environment characterized by a complete disruption of ancestral synteny and the gain of new neighbourhood genes. These drastic alterations to the ancestral gene structure and genomic environment can engender the evolution of a radically novel gene if the retrocopy can escape the associated high risk of pseudogenization.

**Figure 3:**
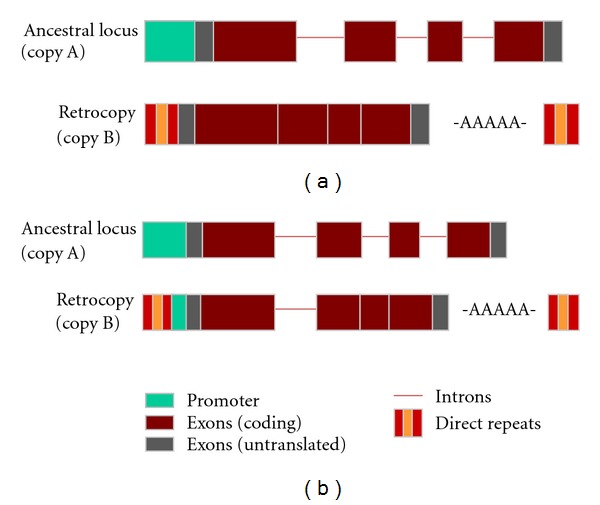
The creation of two classes of retrocopies via RNA-mediated duplication events. (a) Processed copies are created when a spliced mRNA of an ancestral locus is reverse transcribed into cDNA, leading to the creation of a single-exon duplicate from a multiexonic ancestral gene. The processed copy lacks all ancestral introns and the ancestral promoter, while possessing a poly(A) tail at the 3′ end and flanking direct repeats at both the 5′ and 3′ end. (b) A semiprocessed copy can be formed via retrotransposition of a partially-processed pre-mRNA transcript leading to the inheritance of some ancestral introns and flanking region sequence. In the schematic, the retrocopy possesses a poly(A) tail and direct flanking repeats. It lacks introns 2 and 3 but has inherited the ancestral intron 1 and a portion of the ancestral promoter.

The typical outcome of duplication via retrotransposition is thought to be the creation of a single-exon gene duplicate from a multiexonic ancestral gene (Figure 3(a)). Duplication by retrotransposition is implicated by the presence of several diagnostic features in a processed retrocopy, namely (i) the lack of ancestral introns, (ii) an absence of the ancestral upstream promoter region, (iii) coincident boundaries with the ancestral transcribed regions, (iv), a polyadenylation signal followed by a short poly(A) tail at the 3′ end, (v) the presence of flanking direct repeats, and (vi) a novel genomic location. In some instances, retrotransposition of a partially processed pre-mRNA transcript leads to a semiprocessed retrocopy wherein some ancestral introns and flanking region sequence are left intact (Figure 3(b)). Most importantly, these retrocopies can recombine with other duplicated fragments derived from DNA-mediated duplication events or exonize flanking region sequences to create even more drastically altered ORFs. For logistic reasons, I classify these hybrid genes derived from both DNA- and RNA-mediated duplication events as retrocopies though they may need to be categorized as a different class of gene duplicates in the future.

### 4.1. Genomes Vary in the Extent of RNA-Mediated Duplications

RNA-mediated duplication events are certainly common in fly, mammalian, marsupial, and grass genomes as evidenced by the presence of a multitude of retrocopies ([147–152] among others) but are less frequent in birds. *C. elegans* and monotremes [23, 153, 154]. This variation in the frequency of retrogenes among phylogenetically diverse lineages is thought to be contingent on the presence/absence of key enzymes involved in retrotransposition and their activity in the germline in order to enable fidelity of inheritance [154]. A study of 290 evolutionarily young *C. elegans* gene duplicates identified a mere three duplicate pairs wherein one paralog lacked intron(s) relative to the other copy [76]. In a subsequent study, two of these three cases were confirmed as having originated via retrotransposition whereas one case represented intron gain by the derived copy [23], suggesting that 99.3 and 0.7% of paralogs belonging to small gene families in *C. elegans* owe their origins to DNA- and RNA-mediated duplication events, respectively. In *D. pseudoobscura*, 37% of *complete *duplicates were classified as possibly originating via retrotransposition or ambiguous events [77]. RNA-mediated duplication events were implicated in the creation of approximately 7% of evolutionarily young *D. melanogaster* paralogs, and in ~10% of all duplication events in the *D. melanogaster* species complex [24].

### 4.2. Rapidly Emerging Data Highlights the Immense Evolutionary Potential of Retrocopies

 A recognition of the evolutionary potential of retrogenes derived from RNA-mediated duplication events has been slow in coming despite the fact that they, akin to structurally heterogeneous DNA duplicates, can facilitate the creation of novel genes with radically altered ORFs. Lead proponents who have championed the importance of DNA-mediated *partial* and *chimeric* duplications in generating raw material for the origin of novel biochemical functions have been far more skeptical about the importance of retrocopies in evolution. Patthy [75] professed it very unlikely that processed genes (retrocopies) have much to contribute to the origin of novel genes, given that their chance of survival is severely diminished due to the drastic loss of regulatory features at birth. As such, retrocopies were systematically referred to as processed pseudogenes or retropseudogenes [155, 156].

 The last decade has seen the identification of numerous functional retrogenes in diverse lineages. Detailed sequence and functional analyses of these retrogenes have demonstrated them as remarkably adept at recruiting novel regulatory elements and other genic fragments to emerge as mosaic genes conferring a myriad of novel functions. The function of these retrogenes has been best studied in the *Drosophila* lineage and there are several excellent reviews that provide detailed information about their origins and trajectories leading to functional diversification [132, 136, 154, 157, 158]. Rather, I will highlight a few examples that represent the diversity of mechanisms that enable retrocopies to persevere in genomes and evolve novel functions. Retrogenes exhibit a spectrum in the degree of functional divergence from their ancestral gene sources. At one end of the spectrum, some retrogenes evolve to function in a capacity similar to the ancestral gene, with relatively minor modifications to their spatial and temporal expression patterns despite gain of novel exons and promoters from their new genomic environment. At the opposing end of the spectrum and more in line with biological expectations, certain retrogenes gain drastically altered biological functions that appear wholly unrelated to that of their ancestral counterparts.

Two examples presented below represent retrogenes created “functional on arrival” due to the fortuitous inheritance of ancestral promoters during retrotransposition. The murine preproinsulin I gene is derived from the ancestral preproinsulin II which houses one intron in the 5′ untranslated region and another within the coding region. Preproinsulin I is an example of a semiprocessed retrocopy (Figure 3(b)) derived from a partially processed pre-mRNA of preproinsulin II that included the intron in the 5′ untranslated region and ancestral upstream regulatory sequence which enabled its expression following integration into a novel genomic location [159]. Another intriguing example is exemplified by the origin of *PGK-2*. The human *PGK-1* (phosphoglycerate kinase) is an ancestral X-linked gene comprising 11 exons and 10 introns that encodes for an enzyme involved in the metabolism of glucose to pyruvate. Its autosomal paralog *PGK-2* is a retrogene lacking all ancestral introns that shows testis-specific expression in the late stages of spermatogenesis [160, 161]. The X-linked *PGK-1* is inactivated in spermatogenic cells prior to meiosis. However, mature spermatozoa need significant amounts of phosphoglycerate kinase to metabolize fructose present in semen. The inactivation of the single X-chromosome in spermatogenic cells before meiosis is thought to have created the need for a functional autosomal gene copy with a capacity for expression in the testis where the X is inactivated, a role that was fulfilled by the random creation of the *PGK-2* retrocopy. Most interestingly, the *PGK-2 *retrocopy was born functional given that it initially included a copy of the ancestral promoter; only later did it evolve a testis-specific promoter [144, 145]. The preservation of *PGK-2* was favoured by selection for a compensatory response to the inactivation of its progenitor copy. This study has instigated widespread research into what appears to be a common phenomenon in mammals [152, 162] and *Drosophila* [163]—the migratory pattern of X-linked housekeeping genes to autosomal locations via retrotransposition in a bid to escape transcriptional inactivation of X-linked genes in the male germline during meiosis under the influence of natural selection [164].

 The gain of novel functions by retrocopies is also facilitated by their commonly observed fusion with existent gene duplicates derived from DNA-mediated duplication events [165, 166]. The chimeric retrogene *jingwei *in *Drosophila tessieri *and* D. yakuba *was created by retrotransposition of the *Adh *gene, with subsequent insertion of the *Adh* retrosequence into the third intron of a duplicate gene *Yande* (derived from a DNA-mediated *complete *duplication of the *Yellow emperor* gene). The insertion of the single-exon *Adh* retrosequence into *Yande *led to the degeneration of *Yande*'s nine terminal exons, and the origin of the novel gene *jingwei *comprising three *Yande* exons and the single-exonic *adh* retrocopy. This new gene functions as a novel dehydrogenase with increased specificity for long-chain alcohol substrates and a narrowed breadth of expression pattern relative to its ancestor *Adh* [167]. *Adh-Twain* in *D. guanche*, *D. madeirensis,* and *D. subobscura* represents another independent evolutionary formation of a novel gene derived from the fusion of an *Adh* retrocopy and an existing paralog of the *GAPDH* gene labeled as *CG9010* [168]. Unlike *jingwei*, *Adh-Twain *does not appear to have had a major shift in its expression pattern, instead displaying a broad expression pattern similar to its ancestor *Adh* [169].

Retrocopy insertion into a novel genomic location and subsequent exonization of noncoding sequence from its new genomic neighbourhood can yield an ORF with the potential to bestow radically novel functions. The formation of the *Rps23* retrogene in mice via this mechanism has conferred increased resistance to the progression of Alzheimer-causing amyloid plaques, a function quite divergent from the ribosomal protein role of its progenitor copy [170].

## 5. Escaping the Tether of Gene Conversion

 Ohno's canonical model of gene duplicate evolution [16] posits that gene duplicates bearing *complete* sequence and functional redundancy gradually accumulate mutations leading to alternative fates of neofunctionalization or nonfunctionalization with eventual loss from the genome. This model of paralog evolution is overly simplistic because we know paralogs to be capable of nonreciprocal recombination with each other via ectopic (interlocus) gene conversion. Gene conversion is a form of concerted evolution wherein a donor sequence converts a homologous recipient sequence over some length of its tract leading to increased sequence homogeneity between the two paralogs. Hence, gene conversion acts as an effective tether constraining sequence and predictably, functional diversification between paralogs. The evolutionary trajectories of gene duplicates subsequent to their formation is thus governed by two opposing forces; sequence divergence by new mutations and repeated erosion of this achieved sequence heterogeneity via gene conversion [135, 171, 172]. Gene conversion is a ubiquitous process leading to sequence homogenization of paralogs across virtually all organisms that have been subject to detailed enquiries, from microbes to vertebrates [103, 124, 172–179].

 Gene conversion has substantial bearing on the functional fate of gene duplicates. Although we currently lack accurate experimental estimates of the rate of spontaneous ectopic gene conversion between paralogs from mutation accumulation lines that are severely bottlenecked each generation to reduce the efficacy of natural selection, the frequent and independent origin of phenotypes associated with gene conversion events in experimentally evolved lines [113] and detectable signatures of gene conversion among genome-wide studies of paralogs [178] certainly implicate a high rate of ectopic gene conversion. Under environmental regimes where an increased gene dosage of an ancestral protein product is beneficial, natural selection is expected to favour the maintenance of a *complete* structural resemblance and sequence homogeneity between paralogs via gene conversion [180–182]. On the flip side, if spontaneous gene conversion events between paralogs occur at an appreciable frequency, how are paralogs able to escape the evolutionary tether of sequence homogenization by gene conversion to achieve neofunctionalized states? We know that with increasing sequence divergence, the frequency of gene conversion between paralogs is expected to taper off, thereby increasing the probability of functional divergence between paralogs [171]. However, how is this threshold of sequence divergence between paralogs ever achieved in the first place under the constant onslaught of gene conversion? This is especially pertinent for duplicates residing in genomic proximity, given substantial evidence that closely-spaced paralogs experience a higher frequency of gene conversion events [177, 178, 183–185].

Walsh [171] was the first to theoretically explore the conundrum of gene duplicate neofunctionalization in the face of gene conversion pressure. He suggested that “terminator mutations” such as large indels, mobile element insertion and translocation of one paralog to a novel genomic location via retrotransposition may provide the necessary break in sequence homology between paralogs to retard the frequency of gene conversion between them. It is apparent from several studies that the movement of one paralog to another chromosome promotes sequence divergence between the two copies [146] though it is not clear whether this is derived from reduced gene conversion pressure or the inheritance of a novel genomic environment by the paralog. More recently, Innan explored the role of diversifying natural selection in the maintenance of paralog sequence diversity under the pressure of gene conversion [172]. The patterns of DNA variation in human antigen-coding paralogs *RHCE* and *RHD* appear consistent with a model of selection maintaining antigen diversity despite frequent gene conversion, although the strength of selection required to counterbalance homogenization by gene conversion was inferred to be extremely high. Deeb et al. [103] found that despite frequent gene conversion between the X-linked red and green opsin paralogs of Old World primates, certain codons coding for amino acid residues implicated in the separation of peak absorbance between the two pigments were left intact within each paralog thereby implying a role of natural selection in counterbalancing gene conversion.

**Figure 4:**
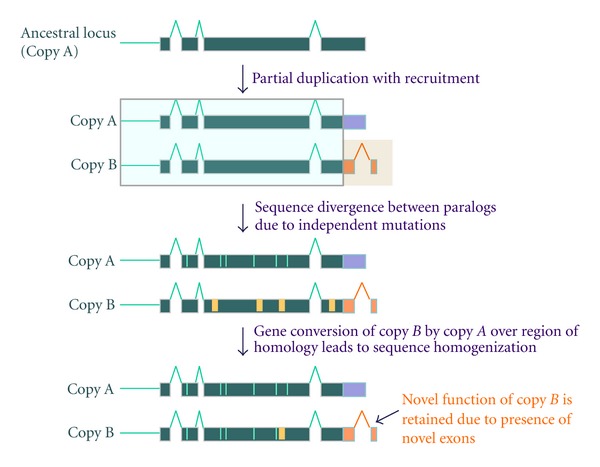
Schematic outlining how structurally heterogeneous duplicates can neofunctionalize by escaping gene conversion based on the *fog-2/ftr-1* case in *C. elegans *[113, 124, 185]. Consider an ancestral gene (Copy A) comprising four exons (top panel) that is partially duplicated. Small shaded rectangles represent exons. The duplication event is initiated in the 5′ flanking region and terminates within exon 4. The large transparent blue rectangle highlights the region of homology between the paralogs as do like coloured exons and introns. The derived copy B additionally recruits noncoding sequence from its genomic neighbourhood to fashion a novel partial exon 4, intron 4 and exon 5 that bears no sequence homology to ancestral copy A (depicted by orange rectangles and highlighted by a large transparent cream rectangle). This novel recruited region also imparts a novel function to copy B. The two paralogs diverge by gradual accumulation of mutations (horizontal narrow lines within exons delineate point mutations). Recurrent episodes of gene conversion of copy B (recipient) by copy A (donor) constrains sequence divergence across the region of homology. Despite high gene conversion pressure, copy B is able to preserve its neofunctionalized state due to the presence of nonhomologous coding sequence that remains unconstrained by gene conversion.

 I additionally suggest that structural heterogeneity among paralogs inherited at birth (as in *partial* and *chimeric* duplicates and retrocopies) plays a very important role in restricting complete homogenization of paralogs via gene conversion, thereby promoting neofunctionalization in addition to the fact that these novel sequences encode novel amino acids. If the unique coding regions in one or both paralog(s) encode novel functional domains, neofunctionalization could be promoted despite ongoing gene conversion in their homologous regions (Figure 4). As such, the creation of a structurally heterogeneous paralog by gene duplication immediately confers on the derived copy a “terminator mutation” as envisioned by Walsh [171] that serves to diminish the homogenizing effects of gene conversion. As a case and point, I revisit the creation of the *fog-2* gene in *C. elegans* from an ancestral gene of unknown function, *ftr-1* (discussed earlier in Section 3.3.2). *fog-2*, implicated in the origin of hermaphroditism in *C. elegans* likely originated from a *partial duplication with recruitment* event resulting from the incomplete duplication of *ftr-1* that prematurely terminated in the terminal exon of *ftr-1*, and subsequently exonized noncoding sequence from its new genomic neighborhood to complete its ORF [113]. Intriguingly, the recruitment of this unique sequence in the 3′ end of *fog-2* likely facilitated its neofunctionalization after duplication [124]. Frequent gene conversions of *fog-2* by *ftr-1* in both experimentally evolved and wild *C. elegans* populations fail to diminish or compromise the function of *fog-2* in hermaphrodite spermatogenesis, given that the neofunctionalized sequence tract in *fog-2* was created by the exonization of novel noncoding sequence and bears no homology to the *ftr-1 *sequence.

## 6. The Influence of Effective Population Size (*N*_*e*_)

Population-genetic theory predicts that the ultimate fate of a mutation (in our case, duplication), be it eventual fixation or loss in a population, depends on the efficacy of natural selection. The efficacy of natural selection (or selection intensity), in turn, depends on the product of (i) the selection coefficient (*s*) of the mutation (also known as the fitness effect of a mutation) and (ii) the effective population size (*N*_*e*_) of the species. Therefore, the intensity of effectiveness of selection is expressed as *N*_*e*_ *s* [186–189]. A decreased intensity of selection could therefore result from either a smaller *s* or a decreased *N*_*e*_. As such, the genomes of prokaryotes and unicellular eukaryotes with extremely large *N*_*e*_ are expected to experience far greater efficacy of selection than those of multicellular eukaryotes with significantly smaller *N*_*e*_. The disparity in *N*_*e*_ across the transitions from prokaryotes to unicellular eukaryotes to multicellular eukaryotes can span several orders of magnitude, from >10^7^ for prokaryotic species and ~10^4^ for larger vertebrates (Table 3).

Lynch and colleagues have posited the provocative hypothesis that the historically lower *N*_*e*_ of multicellular eukaryotes with their concomitant reduced efficacy of selection have provided a permissive environment for the accumulation of certain key elements of genomic architecture that would otherwise be eliminated in genomes more effectively patrolled by purifying selection [81, 190, 191]. Extending this argument, it may be hypothesized that the longer persistence time for such initially nonadaptive genetic elements in eukaryotic species with small *N*_*e*_ could enhance the probability of future exaptation to novel biochemical functions at a later evolutionary stage, leading to the emergence of biological complexity from initially nonadaptive processes.

Given the accumulating evidence that structurally heterogeneous gene duplicates (*partial* and *chimeric*) as well as retrogenes can confer radically novel functions, their near absence in the sequenced genomes of species with large *N*_*e*_ such as *S. cerevisiae* remains a puzzle. Undoubtedly, partially duplicated fragments are less likely to originate in small, compact genomes with shorter genes and fewer, smaller introns. However, spontaneous segmental duplications do originate frequently in yeast [37] and because the gene duplication process appears largely irreverent to gene boundaries, terminal loci within a segmental duplicate fragment should have a higher probability of being partially duplicated. Most gene duplicates may be slightly deleterious when born and likely confer a slight penalty on the fitness of their carriers by creating a minor dosage imbalance. Given the large *N*_*e*_ in microorganisms and unicellular eukaryotes, gene duplicates bearing even slightly negative selective coefficients may be efficiently purged from these genomes. *Partial* duplicates may initially be at a greater selective disadvantage than *complete* duplicates given that the majority of them likely originate lacking function, and are therefore more prone to eradication in these genomes. And might their efficient eradication impose limits to future phenotypic evolution in these species?

These nonadaptive hypotheses certainly warrant further testing as a null model before invoking the ubiquitous guidance of natural selection in the origin of adaptive phenotypes via gene duplication. This is not to say that gene duplicates bearing a great selective advantage at birth are not existent. Like any other class of mutation, gene duplicates can be born advantageous, neutral or deleterious. However, collectively speaking, do different structural classes vary with respect to their fitness effects and how might this, in conjunction with the species *N*_*e*_, impinge on their future evolutionary trajectories? As a first step, mutation accumulation (MA) lines subjected to attenuated selection via repeated bottlenecking provide the best means to investigate the spontaneous rates of occurrence of different structural classes of gene duplicates within phylogenetically diverse genomes and infer the evolutionary forces that govern their subsequent preservation or demise. As discussed earlier, the paucity of SSD-originated *partial* and *chimeric *duplicates in the first yeast genome to be sequenced could be due to lower rates of origin and/or higher probabilities of eradication via natural selection. The characterization of gene duplicates arising in yeast MA lines evolved under conditions of reduced efficacy of selection would enable an accurate determination of the spontaneous rates of origin of different structural classes of gene duplicates. If *partial* and *chimeric *duplicates occur at a significantly higher frequency in the MA lines relative to sequenced genomes not subjected to MA treatment, it would provide evidence for eradication of such duplicates via purifying selection in the latter. We could additionally infer that structurally heterogeneous classes of duplicates, collectively speaking, are more likely to be deleterious relative to *complete* duplicates. In the case of *C. elegans*, the frequency spectrum of structurally homogeneous (*complete* duplicates) and heterogeneous (pooled *partial* and *chimeric*) duplicates in the genomes of MA lines is remarkably concordant with that of the originally sequenced N2 strain [38, 76]. There was an absolute absence of detectable *chimeric* duplicates in the MA lines but this likely reflects the limited diagnostic ability of array Comparative Genomic Hybridization (aCGH) techniques to detect *chimeric* duplicates. The concordant frequencies of these structural classes in the MA lines and the N2 strain strongly suggests that *partial*/*chimeric *duplicates are not subject to greater purifying selection in the *C. elegans *genome, given the relatively low *N*_*e*_ for this species (Table 3).

## 7. Conclusions

The plethora of genomic sequence data has facilitated tremendous advances in our understanding of the gene duplication process. The high frequencies of structurally heterogeneous gene duplicates in many lineages bear direct testament to the inherent promiscuity of the gene duplication process and contribute directly to its potential for rapidly generating novel genes implicated in the emergence of biological innovations. The identification of these structurally heterogeneous duplicates with known novel functions additionally demonstrates that Ohno's canonical model of gene duplicate evolution only represents one of multiple routes that can be assumed by gene duplicates during their evolution. Future investigations should focus on elucidating the relative roles of selection versus random genetic drift in the evolution of new genes via duplication. More importantly, we need to further investigate how the degree of structural resemblance between duplicates and their progenitors impinges on their evolutionary constraints and opportunities in evolution. *Complete* duplicates by virtue of their structural similarity to ancestral genes may be bound to function within the phenotypic bounds of their ancestral counterparts. In contrast, retrocopies and *partial* and *chimeric *duplicates, although more likely to be nonfunctional at birth, may bear greater potential to assume radically novel functions due to the inheritance of novel coding and regulatory elements.

The detailed structural characterization of extant paralogs across phylogenetically diverse genomes would serve to elucidate (i) the various mutational mechanisms responsible for the creation of gene duplicates, (ii) the relative abundance of different structural classes of gene duplicates, (iii) the relative contribution of diverse genomic sequences to the creation of novel genes, (iv) the relative survivorship of different classes of gene duplicates across different age-cohorts of gene duplicates and in different genomic backgrounds, and (v) whether these patterns vary across taxa or display phylogenetic independence.

## Figures and Tables

**Table 1:** Extant percentage of *complete*, *partial* and *chimeric* duplicates originating from small-scale DNA-mediated duplication events in sequenced genomes, mutation accumulation lines, or natural isolates of model organisms.

Phylogenetic distribution	% Gene duplicates from SSD-events			Comments	References
	*Complete*	*Partial*	*Chimeric*		
*Caenorhabditis elegans *					
Sequenced genome of N2 strain	39	21	40	290 duplicate pairs with *dS* ≤ 0.10	[76]
Mutation accumulation (MA) lines	37	63	—	30 spontaneous duplication events across ten MA lines bottlenecked for an average of 432 generations	[38]
*Drosophila melanogaster*					
Sequenced genome	41	27	32	72 duplicate pairs with >80% sequence identity	[24]
Natural isofemale lines	14	76	10	928 CNVs across 15 natural isolates	[45]
*Drosophila pseudoobscura*					
Sequenced genome	44	56	—	101 duplicate pairs derived from DNA-mediated duplication events; >80% sequence identity	[77]
*Saccharomyces cerevisiae*					
Sequenced genome	89	7	4	47 duplicate pairs derived from small-scale duplication events; *dS* ≤ 0.35	[78]

**Table 2:** Some examples of *partial* gene duplicates conferring novel function.

Phylogenetic distribution	Partial duplicate	Ancestral locus	Type of partial duplication	Comments	References
Antarctic Notothenioid fish *Dissostichus mawsoni *	*AFGP *	Trypsinogen	Partial duplication with internal amplification	Creation of a novel antifreeze glycoprotein from an ancestral pancreatic enzyme	[21]
Antarctic eelpout *Lycodichthys dearborni *	*AFPIII *	Sialic acid synthase	Partial duplication in tandem array	Creation of a novel antifreeze protein from an ancestral cytoplasmic enzyme	[110]
*Caenorhabditis elegans *	*fog-2 *	*ftr-1 *	Partial duplication with recruitment	Creation of a novel gene implicated in hermaphrodite spermatogenesis from an ancestral gene of unknown function; evolution of hermaphroditism	[113]
Common ancestor, of *Drosophila simulans*, *D. mauritiana, D. sechellia *	*Hun *	*Bällchen *	Partial duplication with recruitment	Creation of a novel gene with testis-specific expression from an ancestral kinase gene	[109]
*Homo sapiens *	*SRGAP2C *	*SRGAP2 *	Partial duplication	Novel gene unique to humans; linked to increased cognitive ability in the *Homo* lineage	[114, 115]
*Xenopus laevis *	*DM-W *	*DMRT-1 *	Partial duplication	Creation of a novel female sex-determination gene	[116–118]

**Table 3:** Estimates of effective population size (*N*_*e*_) for a sampling of species.

Species	*N*_*e*_	References
Prokaryotes		
*Escherichia coli *	25,000,000	[192]
Unicellular Eukaryotes		
*Paramecium species *	25,000,000–75,000,000	[193]
*Plasmodium falciparum *	210,000–300,000	[194]
*Saccharomyces paradoxus *	10,000,000	[195]
Multicellular Eukaryotes		
Invertebrates		
*Caenorhabditis elegans *	80,000	[196]
*Caenorhabditis remanei *	1,600,000	[197]
*Drosophila melanogaster *	1,150,000	[198]
*Drosophila simulans *	2,600,000	[199]
Plants		
*Arabidopsis lyrata *	138,000	[200]
*Arabidopsis thaliana *	127,000	[200]
*Capsella grandiflora *	500,000	[201]
*Helianthus annuus *	832,000	[200]
*Helianthus petiolaris *	733,000	[200]
European aspen *Populus tremula *	118,000–500,000	[202, 203]
*Zea mays *	590,000	[200]
Vertebrates		
*Mus domesticus *	161,000	[199]
*Mus castaneus *	500,000	[204]
Bonobos	12,300	[205]
Chimpanzee	21,300	[205]
Human	10,400	[205]
Gray Whale	34,410	[206]
